# Regional reemergence of a SARS-CoV-2 Delta lineage amid an Omicron wave detected by wastewater sequencing

**DOI:** 10.1038/s41598-023-44500-0

**Published:** 2023-10-19

**Authors:** Auke Haver, Rick Theijn, Ivo D. Grift, Gino Raaijmakers, Elsa Poorter, Jeroen F. J. Laros, Jaap T. van Dissel, Willemijn J. Lodder

**Affiliations:** 1 Centre for Infectious Disease Control (CIb), National Institute for Public Health and the Environment (RIVM), Bilthoven, The Netherlands; 2 Department of Human Genetics (HG), Leiden University Medical Center (LUMC), Leiden, The Netherlands; 3 Department of BioInformatics and Computational Services (BIR), National Institute for Public Health and the Environment (RIVM), Bilthoven, The Netherlands; 4 Department of Infectious Diseases, Leiden University Medical Center (LUMC), Leiden, The Netherlands

**Keywords:** Next-generation sequencing, Targeted resequencing

## Abstract

The implementation and integration of wastewater-based epidemiology constitutes a valuable addition to existing pathogen surveillance systems, such as clinical surveillance for SARS-CoV-2. In the Netherlands, SARS-CoV-2 variant circulation is monitored by performing whole-genome sequencing on wastewater samples. In this manuscript, we describe the detection of an AY.43 lineage (Delta variant) amid a period of BA.5 (Omicron variant) dominance in wastewater samples from two wastewater treatment plants (WWTPs) during the months of August and September of 2022. Our results describe a temporary emergence, which was absent in samples from other WWTPs, and which coincided with peaks in viral load. We show how these lineage estimates can be traced back to lineage-specific substitution patterns. The absence of this variant from reported clinical data, but high associated viral loads suggest cryptic transmission. Our findings highlight the additional value of wastewater surveillance for generating insights into circulating pathogens.

## Introduction

The ongoing COVID-19 pandemic has promoted the implementation and subsequent integration of wastewater-based epidemiology (WBE) into existing public health surveillance systems^1^. Coronavirus infected individuals shed viral particles in their feces, and the detection of SARS-CoV-2 RNA in wastewater constitutes a non-invasive monitoring tool of virus circulation in the population of a specific region, which is independent of health-seeking behavior^2^. In the Netherlands more than 99% of the population is connected to a wastewater treatment plant (WWTP). Currently, the Dutch National Wastewater Surveillance program samples each WWTP (over 300) four times per week^3^. Molecular analysis is performed on each wastewater sample to determine viral loads per WWTP, which are reported via the Dutch Coronavirus Dashboard^4^. Furthermore, amplicon-based whole-genome sequencing (WGS) is performed on a subset of samples, which allows for additional information on the circulating variants in a population connected to a specific WWTP^5^. The data generated by wastewater surveillance adds to existing surveillance systems such as epidemiological and clinical surveillance, because it provides information on virus circulation among the population, filling in the lowest domain of the disease pyramid and thus provides insight of the overall impact of disease^6,7^.

Whereas consensus sequences are used in clinical testing to determine which variant caused the infection of an individual, an alternate approach is required for sequence data obtained from wastewater samples, which are presumed to contain a heterogenous mix of variants^8^. Indeed, most studies performing WGS employ variant-calling algorithms such as VarScan^9^, LoFreq^10^ or iVar^11^, to identify signature mutations^12–14^. These are subsequently reported or thereafter used for the estimation of relative frequencies of (sub-)lineages^15–17^. These studies have shown high similarity to sequence data of clinical samples in estimation of regional lineage prevalence, as well as the ability to detect lineages not observed through clinical sampling^14,16^.

We report on the detection of a regional and transient reemergence of an AY.43 Delta-variant lineage in two WWTPs during August and September of 2022 amid an Omicron wave. Our results show a transient mutation pattern coinciding with peaks in viral load in WWTP Weert and WWTP Eindhoven located in the South-East of the Netherlands. Notably, the detection of this lineage and its associated sub-lineage in wastewater is absent in the clinical surveillance reporting from the same time period. This observation reaffirms that wastewater surveillance, as a supplement to clinical surveillance, can generate additional information on circulating coronavirus variants.

## Results

### Lineage abundances show transient pattern

**Figure 1:**
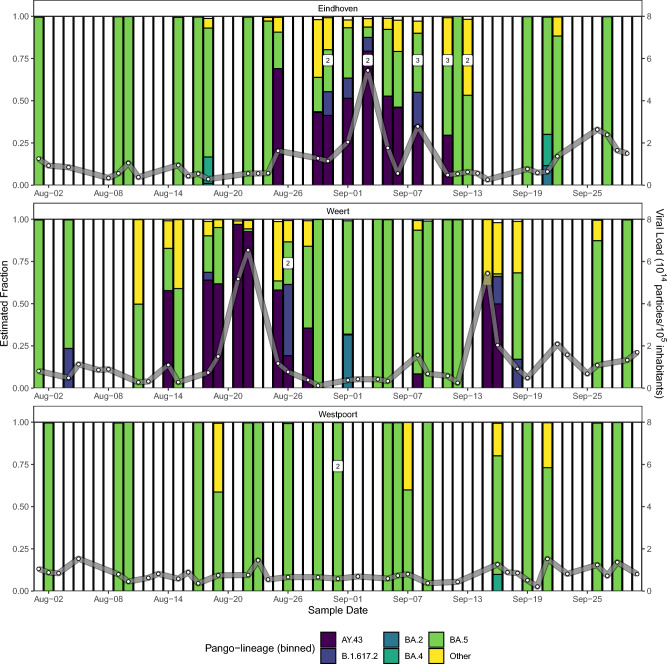
A barplot of estimated fractions (left *y*-axis) of binned pango-lineages for three WWTPS: Eindhoven (Top), Weert (Center) and Westpoort (bottom). Lineages were binned into either the monophyletic clades AY.43 (B.1.617.2.43), BA.2 (B.1.1.529.2), BA.4 (B.1.1.529.4) and BA.5 (B.1.1.529.5) or the paraphyletic B.1.617.2 (excluding AY.43), with the remainder binned into ‘Other’. As a result of the abundance cut-off, not all abundances sum to 1. Viral load estimates (right *y*-axis), corrected for flow and number of connected inhabitant equivalents, are depicted as white dots connected by a gray line. Abundance estimates with bootstrapping confidence intervals are shown in Supplementary Table 1. Samples which were sequenced more than once are labeled with the corresponding number of times.

Initial detection of a B.1.617.2 Delta lineage occurred in a sample from WTTP Weert from August 4th, 2022 (Fig. 1). After detection of mutation patterns of an AY.43 Delta lineage in both a sample originating from WWTP Weert from August 14th, 2022, and one week later in a sample originating from WWTP Eindhoven (August 25th, 2022), the emergence of a regional pattern became apparent. Consequently, these and neighboring WWTPs were subjected to additional sequencing and analysis in order to track the dynamics of these mutation patterns over time. However, detection of Delta lineages was restricted to these two WWTPs in the months of August and September. In total, 50 samples from these two locations were sequenced, consisting of 24 samples for WWTP Eindhoven and 26 samples for WWTP Weert. These WWTPs treat the wastewater of respectively 467,169 and 67,890 inhabitant equivalents^18^. To serve as a reference for wastewater samples in the Netherlands and show variability between wastewater samples, 20 samples from WWTP Westpoort were included in the analysis as well. This location was chosen as it is the fifth largest WWTP in the Netherlands and services 313,928 inhabitant equivalents. Three samples were omitted from the results due to low sequencing coverage (1) and recurrent errors during bootstrapping (2) (Supplementary Table 1).

Lineage abundance estimates revealed a pattern of three transient detections with associated peaks in viral loads (Fig. 1). Whereas multiple Delta lineages were detected, the AY.43 lineage and associated sublineages were observed most frequently (Supplementary Table 1). After the initial detections in WWTP Weert, viral loads steadily increased from August 18th onwards, reaching its highest viral load on August 22nd and subsequently decreasing from August 25th onwards. Between August 29th and September 5th, no Delta lineages were observed in the samples from WWTP Weert. During this period Delta lineages were increasingly found in wastewater samples originating from WWTP Eindhoven, with the highest frequency as well as highest viral load detected in a sample from September 3rd and the last detection in a sample from September 11th. A second and final pattern in Delta lineage detection and viral load increase was observed in the samples from September 15th, 16th and 18th. In addition to Omicron and Delta lineages, the presence of other lineages was estimated as well, however these did not display a similar pattern of detection (Supplementary Table 1). A comparison of these findings to the results of pathogen surveillance data from SARS-CoV-2 test samples, as reported by the RIVM^19^, revealed none of the clinical samples analyzed during this time contained a Delta lineage (Supplementary Fig. 1). Furthermore, after consultation with the local municipal health services, no clear event could be identified to explain this detection pattern.

### Mutation patterns confirm abundance estimates

Estimates of relative lineage abundances could be susceptible to low sequencing coverage and amplification bias^20^. Since the method employed for abundance estimation uses a weighted least absolute deviation (WLAD) regression model^16^, if observed frequencies of defining substitutions vary heavily within individual lineages, the residuals of this model will increase. Accordingly, in order to confirm the validity of the estimates, the residuals for the WLAD model were compared between samples with an estimate of a Delta (B.1.617.2) lineage at a frequency of ≥ 0.001 (Δ^+^) and samples without (Δ^−^) (Supplementary Fig. 2). A significant difference in (Mann–Whitney U-test, *p* < 0.001*)* residuals between the two groups prompted further analysis of the substitution patterns.

**Figure 2:**
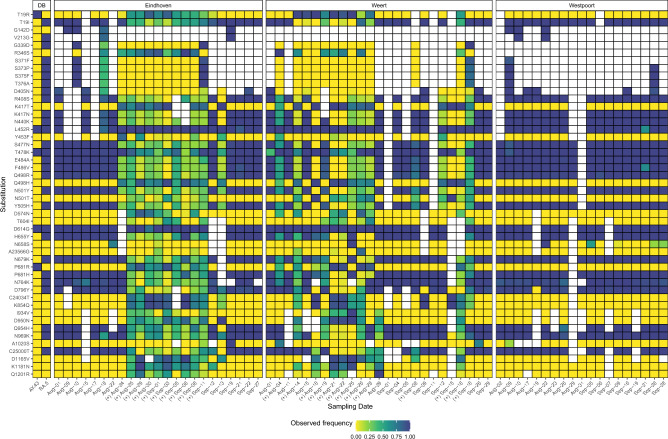
A heatmap of Spike Gene substitution frequencies. Samples (*x*-axis) are split by location (WWTP Eindhoven, Weert and Westpoort) and variant definitions (DB). Substitutions (*y*-axis) were included based on the following criteria: (1) an observed frequency of 0.5 in at least one sample, (2) an observed frequency of. 01 in at least five samples and (3) a *p*-value ≤ 0.05 for variant calling. Spike gene substitutions are shared between lineages BA.5 and BA.4, as well as between B.1.617.2 and AY.34 in the database used (Supplementary Table 2). Only sites with a coverage of at least 10 reads are included. This figure was generated with a custom script written in *R*^22^ (*v4.3.1*) using the ggplot2^23^ (*v3.4.2*) package. (+)Sample with B.1.617 / AY.43 detection.

Analysis of substitution patterns was limited to the Spike gene and revealed a distinct structure between Δ^+^ and Δ^−^ samples (Fig. 2). Dominance of the Omicron BA.5 variant during this time period was reflected by a pattern of high substitution frequencies of BA.5 substitutions as well as an absence of structure in non-BA.5 mutations in the Δ^−^ and WWTP Westpoort samples. Conversely, Δ^+^ samples displayed a more heterogeneous substitution frequency pattern in accordance with the fluctuations in estimated lineage frequencies, whereby samples with lower estimated Delta lineage abundances displayed a substitution pattern more similar to Δ^−^ samples (Figs. 1, 2). Although deviation in this pattern was observed for a subset of samples (WWTP Eindhoven August 24th and September 13th, and WWTP Weert August 19th, August 29th and September 12th), such deviation can occur as lineage abundance estimation is based on substitutions in all sequenced regions. A dropout of amplicon 75 occurred frequently in Δ^−^ samples, whereas this region could be sequenced in most Δ^+^ samples, where it lacked BA.5-specific substitutions (G339D, S371F, S373P, S375F, T376A & D405N), which suggests an amplification bias. These differences were similarly present in substitutions not associated with either the BA.5 or AY.43 lineage according to the database used (Supplementary Table 2), such as A23056C (Q498H), C24034T (synonymous), and G24410A (D950N). However, association of substitutions to lineages can vary per database used, as D950N is associated to the Delta clade in the Nextstrain system^21^. Whereas a detailed analysis of private mutations is outside the scope of this work, these patterns confirm the Delta-lineage detections.

## Discussion

In this study, we report on the transient detection of AY.43 substitution patterns obtained by sequencing and consequent bioinformatic analysis of wastewater samples from the WWTPs Eindhoven and Weert. Our data first shows an emergence in WWTP Weert, followed by a transition to WWTP Eindhoven and a transition back to WWTP Weert, after which the AY.43 mutation patterns disappear. Notable is the positive association shown between the AY.43 abundance estimates and the observed viral load.

Whereas many AY.43-specific mutations were found, other mutations were discovered which could not be attributed to either BA.5 or AY.43, which combined with the higher residuals of these demixing models suggest the presence of a cryptic lineage most similar to Delta but possessing unique mutations. Cryptic lineages have been detected in wastewater before^14–16,24^. Westcott et al.^25^ similarly observed a reemergence of a Delta variant in wastewater, isolated at a single WWTP and occurring three times in a timeframe of nine months. Gregory et al.^13^ described that the reservoir for these viruses could be one of three possible sources: (1) one of non-human/animal origin, (2) an asymptomatic group of infected persons or (3) a group of immunocompromised persons. The study by Shafer et al.^26^ described how they were able to track a cryptic lineage back to a group of approximately 30 people and pointed a immunocompromised individual with a persistent gastrointestinal infection out to be the point of transmission. It is still unknown how many infected individuals would be needed to observe the rise in viral load in both locations, as shedding rates vary depending for instance on both the variant and health status of the infected individual^2^. Moreover, in case of the Delta and Omicron variants, reduced shedding has been observed for the latter, which reportedly could be attributed to a lower required infective dose^27^. With no further observations of this AY.43 lineage in wastewater samples or reported from clinical sampling, source attribution remains unknown.

Lastly, the absence of Delta lineages in clinical surveillance would suggest this cryptic transmission to have had little to no clinical impact. However, this does not preclude similar future events to lead to an increased disease burden, in which WBE could help identify signals ahead of clinical sampling. In conclusion, the results shown here confirm the relevance of WGS performed on wastewater samples as a supplement to other surveillance tools, such as clinical surveillance, as it enables the detection of otherwise unobserved virus circulation.

## Methods and materials

### Sample retrieval, preparation and sequencing

Twenty-four-hour composite influent wastewater samples from the WWTPs Eindhoven and Weert, August and September 2022, were selected for WGS analysis. Nucleic acids used to determine the viral load were additionally treated with DNase and purified using RNA Clean and Concentrator columns (Zymo Research, https://www.zymoresearch.com/). On the purified RNA, a cDNA synthesis using random hexamer primers was performed and on the obtained cDNA a PCR was performed using the Artic V4.1 amplicon panel (ARTIC network, https://artic.network). Libraries were prepared with the TruSeq Nano DNA kit (Illumina, https://www.illumina.com). Paired-end short-read sequencing was performed in multiple runs a MiSeq platform (Illumina, https://www.illumina.com) using either combinatorial-dual (CD) or unique-dual (UD) indexes (Illumina, https://www.illumina.com).

### Bioinformatic analysis

Cleanup of sequencing reads was based on the method used in the SARS2seq pipeline (https://github.com/RIVM-bioinformatics/SARS2seq). Software versions and parameters for the various steps are described in Supplementary Table 3. Briefly, adapter trimming was performed by aligning raw sequence reads using minimap2^28^ to the Wuhan reference genome (NC_045512.2) and subsequent removal of soft-clipped bases. Next, sequence quality control was performed with fastp^29^. Lastly, primer sequences were removed using the AmpliGone tool (https://github.com/RIVM-bioinformatics/AmpliGone). Reads of wastewater samples which were subject to multiple rounds of sequencing were combined for further analysis using Samtools^30^. Only libraries with at least 50% genome coverage at a depth of 10 reads were included. Variant calling was performed with the iVar tool (https://github.com/andersen-lab/ivar) using default settings. On the called variants, lineage demixing was performed with the Freyja tool (https://github.com/andersen-lab/Freyja) developed by Karthikeyan et al.^16^, which uses the UShER global phylogenetic tree to determine which substitutions are associated with which lineages. The output of the Freyja tool, including lineage definitions, substitution frequency and genome coverage, was used to analyze substitution frequency with custom scripts in *R*^22^ (v4.3.1). Only sites with coverage ≥ 10 were considered. Substitutions not associated with either the BA.5 or AY.43 lineage were included only when they were found to occur in at least 5 samples with a frequency of ≥ 0.01, in at least 1 sample with a frequency of ≥ 0.50 and with a *p*-value ≤ 0.05 for variant calling. All figures in the manuscript were generated with custom scripts written in *R*^22^ (v4.3.1) using the ggplot2^23^ (v3.4.2) package.

### Supplementary Information

Supplementary Information 1.

Supplementary Information 2.

## Data Availability

Raw sequencing reads are available under Bioproject PRJNA922726. Viral load data is available at https://data.rivm.nl/covid-19/COVID-19_rioolwaterdata.csv.
