# Supplementary material for: Regional reemergence of a SARS-CoV-2 Delta lineage amid an Omicron wave detected by wastewater sequencing
Source: Sci Rep. 2023 Oct 19;13:17870. doi: 10.1038/s41598-023-44500-0 (PMC10587120; doi:10.1038/s41598-023-44500-0)
Supplement: Supplementary file 1 — Supplementary Information 1. [file 41598_2023_44500_MOESM1_ESM.pdf]

**Supplementary information:**

**Title:** Regional reemergence of a SARS-CoV-2 Delta lineage amid an Omicron wave detected by wastewater sequencing

**Authors:** Auke Haver[1,2], Rick Theijn[1], Ivo D. Grift[1], Gino Raaijmakers[1], Elsa Poorter[1], Jeroen F.J. Laros[2,3], Jaap T. van Dissel[1,4], Willemijn J. Lodder[1]*.

**This PDF file includes:**

- Figure S1
- Figure S2
- Table S1
- Table S2
- Table S3

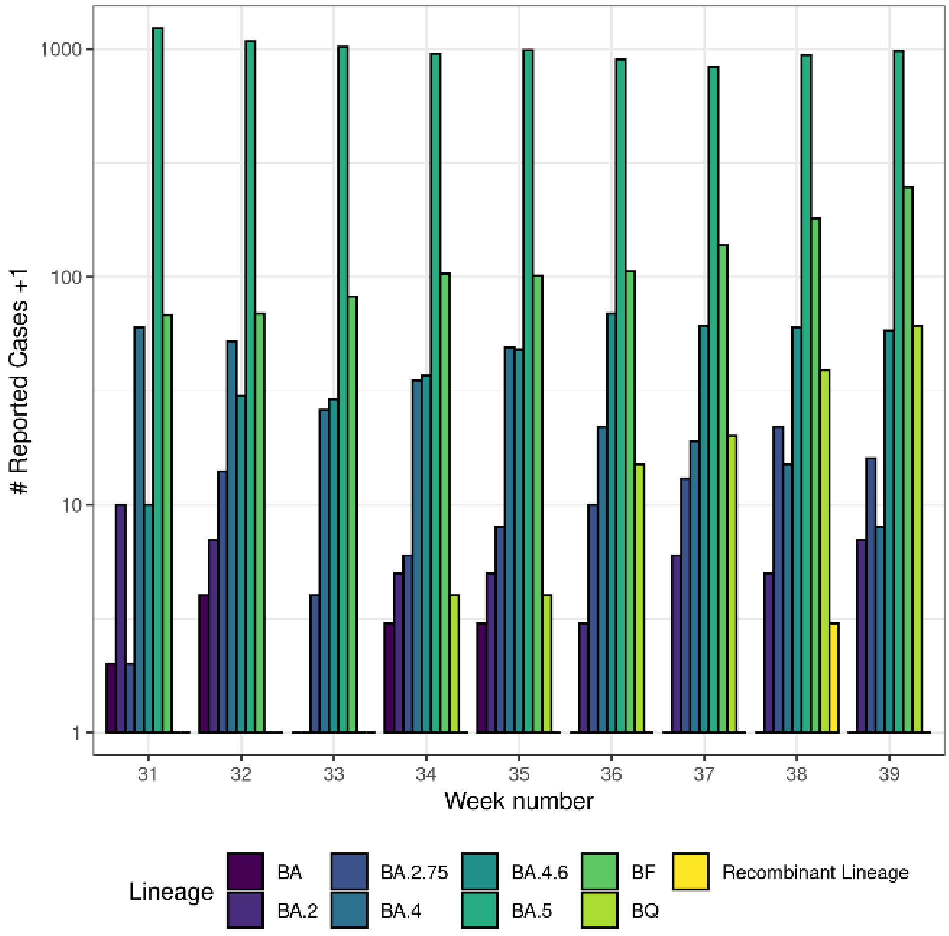


**Supplementary Figure 1:** SARS-CoV-2 variants detected by the pathogen surveillance of the RIVM during the months August and September 2022.

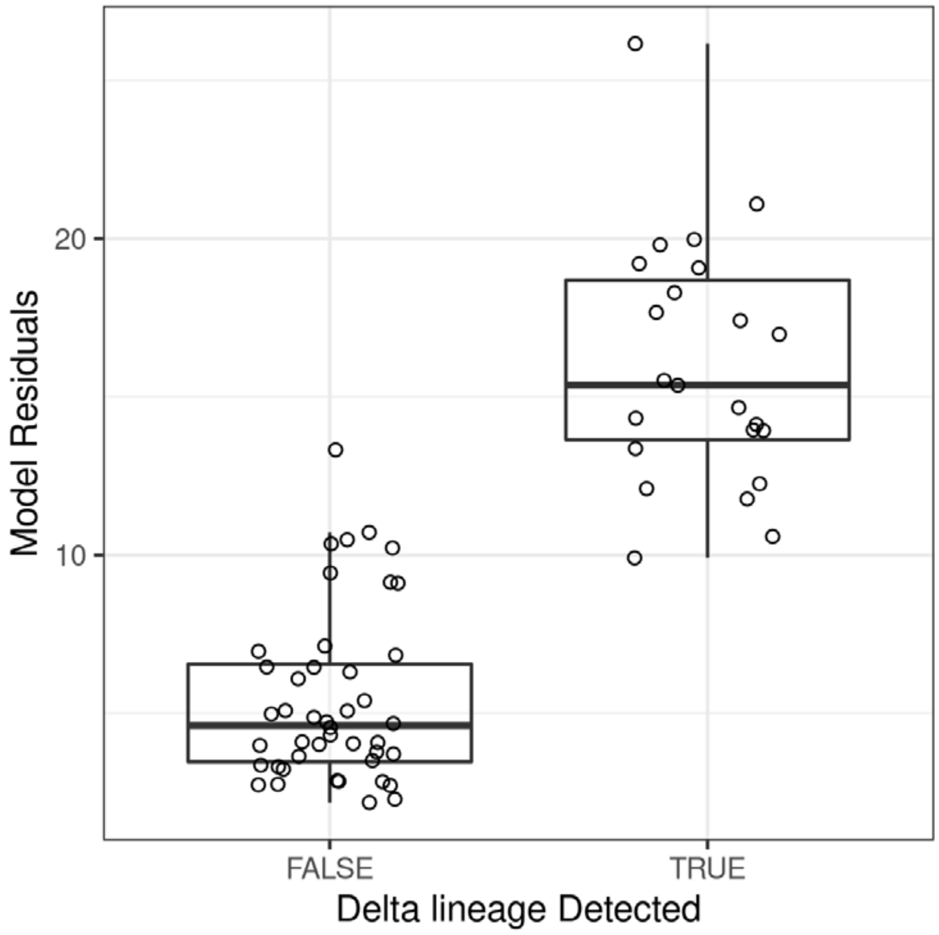


**Supplementary Figure 2:** A barplot of the residuals of the weighted least absolute deviation problem. Structure in residuals (dots) is shown to be different depending on the detection of an AY-lineage.

**Supplementary Table 1: Results of SARS-CoV-2 lineage frequency estimation (Available as separate spreadsheet)**

Samples are shown per wastewater treatment plant (*location*) and date of sampling (*sample_date*). Lineage frequency estimates (*abundance*) were generated using the Freyja[1] software tool. De-aliased lineage names (*lineage_full*) were included for interpretability. Data shown includes the residuals of the regression model (*resid*), the genome coverage at >= 10x depth (*coverage*), the sequence libraries generated and combined for a sample (*n_samples*) and the 0.975 (*upper*) and 0.025 (*lower*) percentiles of the 100-fold bootstrapping estimate.


1. Karthikeyan, S. et al. Wastewater sequencing reveals early cryptic SARS-CoV-2 variant transmission. *Nature*, 609, 101-108; 10.1038/s41586-022-05049-6 (2022).

**Supplementary Table 2: Lineage associated-mutations**

| substitution_nuc | substitution_AA | B.1.617.2 | AY.43 | BA.4 | BA.5 |
|---|---|---|---|---|---|
| C21618G | T19I | 1 | 1 | 0 | 0 |
| C21618T | T19R | 0 | 0 | 1 | 1 |
| T22200G | V213G | 0 | 0 | 1 | 1 |
| G22578A | G339D | 0 | 0 | 1 | 1 |
| C22674T | S371F | 0 | 0 | 1 | 1 |
| T22679C | S373P | 0 | 0 | 1 | 1 |
| C22686T | S375F | 0 | 0 | 1 | 1 |
| A22688G | T376A | 0 | 0 | 1 | 1 |
| G22775A | D405N | 0 | 0 | 1 | 1 |
| A22786C | R408S | 0 | 0 | 1 | 1 |
| G22813T | K417N | 0 | 0 | 1 | 1 |
| T22882G | N440K | 0 | 0 | 1 | 1 |
| T22917G | L452R | 1 | 1 | 1 | 1 |
| G22992A | S477N | 0 | 0 | 1 | 1 |
| C22995A | T478K | 1 | 1 | 1 | 1 |
| A23013C | E484A | 0 | 0 | 1 | 1 |
| T23018G | F486V | 0 | 0 | 1 | 1 |
| A23055G | Q498R | 0 | 0 | 1 | 1 |
| A23063T | N501Y | 0 | 0 | 1 | 1 |
| T23075C | Y505H | 0 | 0 | 1 | 1 |
| A23403G | D614G | 1 | 1 | 1 | 1 |
| C23525T | H655Y | 0 | 0 | 1 | 1 |
| T23599G | N679K | 0 | 0 | 1 | 1 |
| C23604A | P681H | 0 | 0 | 1 | 1 |
| C23604G | P681R | 1 | 1 | 0 | 0 |
| C23854A | N764K | 0 | 0 | 1 | 1 |
| G23948T | D796Y | 0 | 0 | 1 | 1 |
| A24424T | Q954H | 0 | 0 | 1 | 1 |
| T24469A | N969K | 0 | 0 | 1 | 1 |
| C25000T | synonymous | 0 | 0 | 1 | 1 |

## Supplementary Table 3: Parameters for software tools

| Software tool | Version | Usage | Command |
|---|---|---|---|
| fastp | 0.23.2 | QC | fastp --cut_right -M 20 -W 5 -l 100 |
| AmpliGone | 1.1.0 | Primer removal | ampligone --amplicon_type end-to-mid |
| minimap2 | 2.24 | Sequence Alignment | minimap -ax sr |
| Samtools | 1.14 | Convert SAM file to BAM file | samtools view -uS |
| Samtools | 1.14 | Sort BAM file | samtools sort |
| Samtools | 1.14 | Index BAM file | samtools index |
| Samtools | 1.14 | Merge BAM files | samtools merge |
| Samtools | 1.14 | Index reference sequence | samtools faidx |
| Samtools | 1.14 | Sequence pileup | samtools mpileup -aa -A -d 0 -B -f -Q 20 -d 600000 |
| iVar | 1.3.1 | Variant Calling | ivar variants -q 20 -t 0.0 -m 0 |
| Freyja | 1.3.11 | Variant Calling (for demixing) | freyja variants |
| Freyja | 1.3.11 | Demix lineages | freyja demix --confirmedonly --eps 0.001 |
| Freyja | 1.3.11 | Demix lineages with bootstrapping | freyja boot --confirmedonly --eps 0.001 -nb 100 |
